# *Codonopsis pilosula* Polysaccharides Exert Antiviral Effect Through Activating Immune Function in a Macrophage Model of Bovine Viral Diarrhea Virus Infection

**DOI:** 10.3390/vetsci12050415

**Published:** 2025-04-27

**Authors:** Xiaowei Feng, Lei Wang, Jingyan Zhang, Haipeng Feng, Xiaoliang Chen, Junyan Wang, Mingxian Shi, Kang Zhang, Jianxi Li

**Affiliations:** 1 Traditional Chinese Veterinary Technology Innovation Center of Gansu Province, Lanzhou Institute of Husbandry and Pharmaceutical Sciences, Chinese Academy of Agricultural Sciences, Lanzhou 730000, China; 82101231377@caas.cn (X.F.); wanglei03@caas.cn (L.W.); zwzh1223@126.com (J.Z.); fenghp105@126.com (H.F.); 18148345073@163.com (X.C.); WJY620422@163.com (J.W.); shimingxian2020@163.com (M.S.); 2 College of Veterinary Medicine, Hebei Agricultural University, Baoding 071000, China; 3 College of Veterinary Medicine, Gansu Agricultural University, Lanzhou 730000, China

**Keywords:** *Codonopsis pilosula* polysaccharides, bovine viral diarrhea virus, macrophage, antiviral activity, immunomodulatory activity

## Abstract

Bovine viral diarrhea (BVD) is a common viral disease in cattle that causes huge economic losses in naïve herds that are introduced to bovine viral diarrhea virus (BVDV). Currently, there are no available anti-BVDV drugs due to the variety and mutability of strains; therefore, developing new anti-BVDV drugs is of great significance. The aim of this study was to investigate the anti-BVDV effects and immunomodulatory activities of *Codonopsis pilosula* polysaccharides (CPPs) in BVDV-infected bovine macrophage (BoMac) cells. CPPs directly inactivated BVDV particles and intervened in BVDV absorption process. The immunity suppression resulting from BVDV in BoMac cells was restored by CPPs, as was verified by phagocytosis increase, the expression up-regulation of cell surface co-stimulatory molecules (CD40, CD80, and CD86), and antigen-presenting function recovery. Furthermore, the expressions of cytokines including TNF-α, IFN-γ, IL-6, IL-18, IL-1β, Caspase-3, Bim, and Bcl-xL at the mRNA and proteins levels were modulated, and a reduction in the apoptosis rate was observed, which demonstrates that CPPs attenuated inflammation and apoptosis induced by BVDV. Collectively, our findings reveal new pharmacological properties of CPPs, which exert anti-BVDV efficacy and regulate immune injury induced by BVDV in BoMac cells, indicating that CPPs are a potential option for BVDV prevention in clinical application.

## Simple Summary

Bovine viral diarrhea is an infectious disease caused by bovine viral diarrhea virus. The immunosuppression caused by this disease poses a serious threat to the cattle industry and, so far, there is no effective treatment for bovine viral diarrhea virus infection. Therefore, developing new anti-bovine viral diarrhea virus drugs is of great significance. This aim of this study was to investigate the antiviral and immunomodulatory activities of *Codonopsis pilosula* polysaccharides using bovine macrophages. We demonstrate that the anti-bovine viral diarrhea virus effects of *Codonopsis pilosula* polysaccharides against bovine viral diarrhea virus were mainly inhibition of viral adsorption and direct virucidal activity, with the former exhibiting predominant efficacy. Furthermore, our results show that *Codonopsis pilosula* polysaccharides could enhance cellular phagocytosis and antigen presentation, attenuate the expression of bovine viral diarrhea virus-induced inflammation and apoptosis-associated factors, and reduce the rate of apoptosis in bovine macrophages. These results suggest that *Codonopsis pilosula* polysaccharides could activate the antiviral response through immunomodulatory action and play a special role in blocking virus adsorption. In conclusion, our results suggest that *Codonopsis pilosula* polysaccharides have potential as novel therapeutic and immunomodulatory drugs for the prevention and control of bovine viral diarrhea virus infection.

## 1. Introduction

Bovine viral diarrhea (BVD), also known as mucosal disease, can cause immunosuppression, respiratory disease, and reproductive disturbance in cattle herds that are acutely infected with bovine viral diarrhea virus (BVDV) or suffering a chronic epidemic. The main sources of the infection include secretions, excreta, saliva, and blood from sick cattle. The clinic characteristics are mucous membrane inflammation, erosion, necrosis, and diarrhea. This disease has the characteristics of high incidence, wide distribution, and rapid spread both domestically and internationally [1,2]. BVDV, a member of the *Flaviviridae* family and genus *Pestivirus*, is one of the key pathogens responsible for the disease [3]. Once the virus infects the host, BVDV can cause persistent infection and immunosuppression, severely impeding the progress of animal husbandry. Currently, vaccine immunization remains the primary clinical measure for the prevention of BVD. However, the effectiveness of current prevention and control efforts remains limited due to the diversity of virus strains, frequent mutation, and high infection rate [4]. Therefore, finding more effective measures to prevent and control BVD is an urgent issue in the industry [5].

BVDV infection interferes with the innate and adaptive immune response after invading the host and undergoes immune system evasion, which triggers an inflammatory response and apoptosis [6]. Previous studies have confirmed that BVDV infection can directly destroy the normal immune function of various immune cells, including neutrophils, monocytes, macrophages, and dendritic cells [7,8], and at the same time activate monocytes and macrophages to secrete a large number of immune regulatory factors that regulate immune response, except for phagocytosis and antigen presenting cells [9]. Furthermore, BVDV infection can reduce the phagocytic capabilities of macrophages and down-regulate the expression levels of immunoglobulin and chemokines. As a result, animals that have been infected with BVDV are more susceptible to secondary infections by other pathogenic microorganisms [10]. Therefore, further investigation on immunotherapy drugs to prevent BVDV infection and improve immunity and health in cattle is needed, which is also of great significance for reducing the economic losses caused by BVDV infection in the cattle industry.

*Codonopsis pilosula* (CP) is a famous Chinese herbal medicine known for its abilities to tonify qi, strengthen the spleen, and nourish blood [11]. *Codonopsis pilosula* polysaccharides (CPPs), a primary component of CP, comprise glucose, fructose, inulin, and sucrose [12,13], showing multiple pharmacological effects such as antioxidant, immune regulatory, antibacterial, and antiviral properties [14,15,16,17,18]. It was pointed out that CPPs have antiviral effects on the duck hepatitis virus and the Newcastle disease virus [19,20]. Meanwhile, CPPs can stimulate immune cells to produce immunomodulators, thereby activating cellular immune response and regulating the expression of inflammatory factors in immunosuppressed mice [21,22]. Although the antiviral activity of CPPs has been reported, the effect of CPPs on BVDV infection remains unresolved.

Therefore, in this study, we examined the anti-BVDV effect of CPPs in a bovine macrophage (BoMac) cell model of BVDV infection. Subsequently, we investigated the effect of CPPs on phagocytic activity and antigen-presenting function, cytokine expression, and apoptosis in cells following infection by BVDV. Our findings highlight the potential value of CPPs against BVDV infection and provide new insights into the mechanism of CPPs to alleviate the immunopathological injury caused by BVDV.

## 2. Materials and Methods

### 2.1. Cells, Virus, and Tested Compound

The bovine macrophage cell line (BoMac), kindly provided by Professor Aizhen Guo (Huazhong Agricultural University in Wuhan, China), was incubated in RPMI 1640 medium (Gibco, Grand Island, NY, USA) supplemented with 1% penicillin/streptomycin (Gibco, NY, USA) and 10% fetal bovine serum (Gibco, NY, USA).

Madin-Darby bovine kidney (MDBK) cells were obtained from the China Institute of Veterinary Drug Control and were cultured in RPMI 1640 medium (Gibco, NY, USA) supplemented with 1% penicillin/streptomycin and 10% fetal bovine serum (Gibco, NY, USA).

The BVDV-NADL standard strain (CP type) used in this study was obtained from the China Veterinary Drug Control Centre (CVCC AV67). The virus was propagated in MDBK cells. Infected MDBK cells and supernatant were repeatedly harvested and freeze-thawed three times. Centrifugation was performed at 2000 r/min for 10 min, and the supernatant was harvested as the viral solution. BoMac cells were seeded in a 96-well plate at a density of 1 × 10^4^ and incubated at 37 °C for 24 h. Tenfold serial dilutions of the virus were inoculated into the cells and incubated at 37 °C. The cells were observed continuously for 7 to 9 days and per-well manifestation of cytopathic effect were recorded. Viral titters were estimated using the Reed–Muench method.

CPPs (number: SR8506) were purchased from Beijing Solaibao Technology Co., Ltd. (Beijing, China). The purity of the CPPs was greater than 90% (UV ≥ 90%) according to HPLC analysis. CPPs (15 mg) were dissolved in 5 mL of serum-free medium, with 3 mg/mL as a stock solution.

### 2.2. Cell Viability Assay

BoMac cells were seeded in a 96-well plate at a density of 1 × 10^4^ and incubated at 37 °C for 24 h, and then added to various concentrations of CPP solutions and incubated for 48 h. Then, 10 μL of CCK-8 (Biosharp, Hefei, China) solution was added to each well and incubated again at 37 °C for 1.5 h. The optical density (OD) was measured at 450 nm using a microplate spectrophotometer (Bio Tek, Winooski, VT, USA). The cell viability was calculated using following formula:

$$Cell\;viability=\frac{OD\;of\;the\;experimental\;group-OD\;of\;the\;blank\;group}{OD\;of\;the\;control\;group-OD\;of\;the\;blank\;group}\times 100\%$$

Note: Experimental group: groups with different concentrations of CPPs; Control group: CONT group; Blank group: blank control group.

### 2.3. Antiviral Effect Assay

CPPs were added at different infection phases to investigate their antiviral activity and mechanism. A BoMac cell monolayer grown on a 6-well plate at density of 3 × 10^5^ was incubated at 37 °C for 24 h, after which BVDV (100 TCID50) was treated with various doses of CPPs under three conditions. (1) The cells were infected with BVDV fluids (100 TCID50) after being treated with various doses of CPP solution for 3 h at 37 °C and were then incubated at 37 °C for 48 h. (2) BVDV inoculum (100 TCID50) was mixed with various concentrations of CPP solution at a ratio of 1:1 (*v*/*v*), incubated at 37 °C for 3 h, and then inoculated into the mixture to cells grown for 48 h. (3) The cells were treated with various doses of CPP solution after being infected with BVDV fluids (100 TCID50) for 3 h at 37 °C, and then cultured at 37 °C for 48 h. Then, the cell morphology and vitality were observed by using an inverted microscope and a CCK-8 kit (Biosharp, Hefei, China), and the expression of BVDV copies and protein were detected by performing RT-qPCR and immunofluorescence, respectively.

### 2.4. Real-Time Quantitative PCR (RT–qPCR)

The virus RNA was extracted from BoMac cells using Ackeri’s Viral RNA Extraction Kit. An absolute quantitative test was carried out using a recombinant plasmid previously constructed in our lab and primers to calculate the number of viral nucleic acid copies in each group of cells based on the standard curve. The primers were used as follows: BVDV-F (5′-3′): GGCATGCCCTTAGTAGGACT, BVDV-R (5′-3′): GCCATGTACAGCAGACAT. The RT-PCR assay was performed under the following conditions: 42 °C for 5 min and 95 °C for 30 s, followed by 45 cycles at 95 °C for 5 s and 60 °C for 30 s.

After being treated with various doses of CPP solution for 3 h at 37 °C, the cells were infected with BVDV fluids (100 TCID50) and then incubated at 37 °C for 48 h. The total RNA was extracted from the cells using Ackeri’s Universal RNA Extraction Kit. A NanoDrop ND-2000 spectrophotometer (Thermo Fisher Scientific, Waltham, MA, USA) was used to determine the RNA quality of all samples. The RevertAid First Strand cDNA Synthesis Kit (Accurate, Changsha, China) was used to synthesize first-strand cDNA. Primer-BLAST software was used to design primers (Table 1). The RT-qPCR was performed on a LightCycler 480 system with a SYBR^®^ Green Premix Pro Taq HS qPCR Kit (Rox Plus) (Accurate, Changsha, China) with an Applied Biosystems 7500 Fast instrument (ABI, Los Angeles, CA, USA). The qPCR conditions were presented as follows: 95 °C for 30 s, followed by 40 cycles of denaturation at 95 °C for 5 s and annealing/elongation for 30 s. Moreover, relative transcript expression levels were calculated via the 2^−△△CT^.

### 2.5. Immunofluorescence Assay

The cells were fixed using 4% paraformaldehyde after washing 3 times with PBS. Then, after being washed 3 times with PBS, the cells were stained using the Fluorescein isothiocyanate (FITC) method of BVDV combined with the fluorescent antibody staining method, followed by staining of the cell nuclei with DAPI. The cells were placed under a laser confocal microscope (ZEISS, Oberkochen, Germany) to observe the positive signal.

### 2.6. Determination of Cell Phagocytic Function

After being treated with various doses of CPP solution for 3 h at 37 °C, the cells were infected with BVDV fluids (100 TCID50) and then incubated at 37 °C for 48 h. The phagocytic activity of the BoMac cells was assessed using sf-GFP labeled *Escherichia coli* (*E. coli*) (BNCC, Beijing, China). Briefly, a phagocytosis assay was performed with sf-GFP-tagged bacteria for 4 h at a multiplicity of infection (MOI) of 1:1. The control cells were cultured as normal, and equal amounts of serum-free medium were added to the cells. After being co-cultured at 37 °C at a 5% CO_2_, non-internalized bacteria along with the control cells were removed from the external surface by washing with PBS and then were observed with a laser confocal microscope (LMS800, ZEISS, Germany).

### 2.7. Determination of the Cellular Antigen-Presenting Function

After being treated with different doses of CPP solution at 37 °C for 3 h, cells were infected with BVDV solution (100 TCID50) and incubated at 37 °C for 48 h. Then, cells were collected by centrifugation at 600× *g* for 8 min and washed once with PBS. Subsequently, 5 μL of antibodies against CD40, CD80, and CD86 (Thermo Fisher Scientific, MA, USA) were added to each group and incubated at 4 °C in the dark for 30 min. Afterwards, cells were washed with PBS again, centrifuged at 600× *g* for 8 min, and finally resuspended in 500 μL PBS. Cell analysis was performed using a flow cytometer (BD, Franklin Lakes, NJ, USA).

### 2.8. Enzyme-Linked Immunosorbent Assay (ELISA)

A bovine ELISA kit (MLBio, Shanghai, China) with a minimum detectable concentration < 0.1 pg/mL was used to assess the pro-inflammatory cytokine content, including IL-6, IL-18, IL-1β, and TNF-α, in the cell supernatant. After being treated with various doses of CPP solution for 3 h at 37 °C, the cells were infected with BVDV fluids (100 TCID50) and then incubated at 37 °C for 48 h. The supernatant was collected by centrifuging at 1000× *g* for 20 min at 4 °C, according to the manufacturer’s instructions. The optical density was measured at 450 nm with a microplate spectrophotometer (Bio Tek, VT, USA).

### 2.9. Cell Apoptosis Assay

Cell apoptosis was measured with an Annexin-V Apoptosis Detection Kit (YESEN, Shanghai, China), according to the manufacturer’s instructions. In brief, after being treated with various doses of CPP solution for 3 h at 37 °C, the cells were infected with BVDV fluids (100 TCID50) and then incubated at 37 °C for 48 h; then, the cells were collected by centrifugation at 600× *g* for 5 min at 4 °C and stained with Annexin-V and PI after being washed with cold PBS. The samples were gently mixed and incubated for 15 min at room temperature in the dark. Then, 400 μL of 1 × Annexin V Binding Buffer was added to each tube and gently mixed, and the number of apoptotic cells was measured by flow cytometer (BD, NJ, USA).

### 2.10. Statistical Analyses

IBM SPSS Statistics 21 and one-way ANOVA were used to analyze the data in this study. Multiple group comparisons were carried out using the LSD method, with the results expressed as mean ± standard error of mean (SEM). All experiments were performed with at least three independent replicates. Graphical representations were completed using GraphPad Prism 8.0. A *p*-value of <0.05 was considered statistically significant.

## 3. Results

### 3.1. Effect of CPPs on Bomac Cells Viability

**Figure 1:**
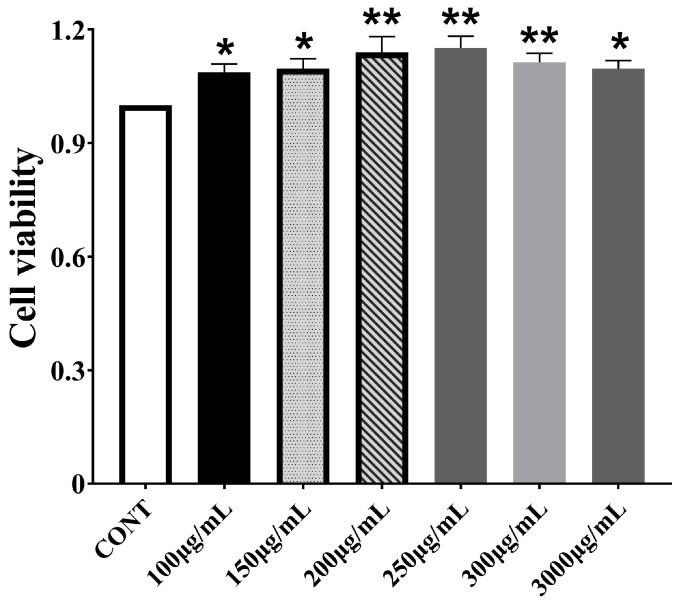
Effect of *Codonopsis pilosula* polysaccharides (CPPs) on the viability of bovine macrophage (BoMac) cells. Data represented are means ± standard errors of means (SEMs) from triplicate experiments. * *p* < 0.05, ** *p* < 0.01.

To determine the safe concentration, CPPs were administered to BoMac cells at various concentrations for 48 h. Based on the CCK-8 assay results, cell viability increased with rising CPP concentration, peaking at 250 μg/mL. Consequently, concentrations of 100 μg/mL, 200 μg/mL, and 300 μg/mL were subsequently selected as the low, medium, and high doses for further tests (Figure 1).

### 3.2. Blocking Effect of CPPs on BVDV Adsorption

**Figure 2:**
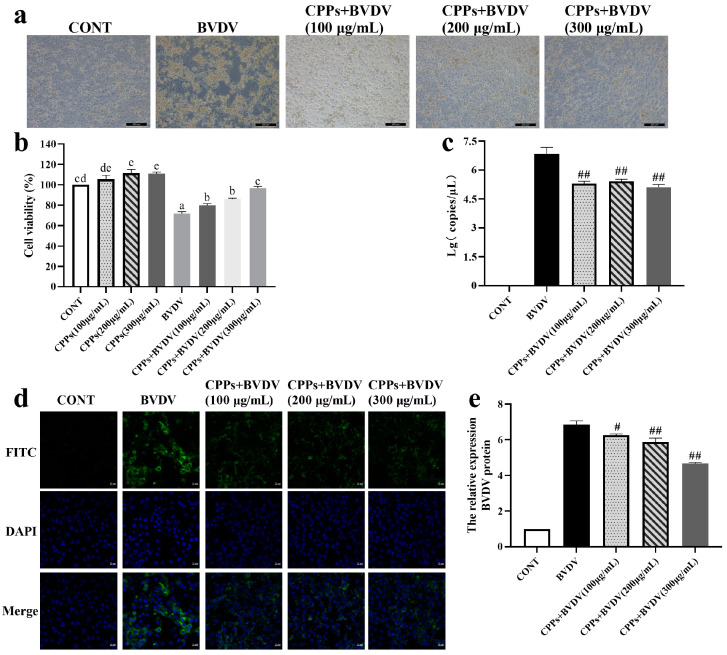
CPP inhibits the adsorption of bovine viral diarrhea virus (BVDV) in BoMac cells. (**a**) Cell morphology changes observed by inverted microscope. (**b**) Cell viability determined by CCK-8 assay. (**c**) BVDV nucleic acid copy number was detected by RT-qPCR. (**d**) BVDV viral protein expression was detected by immunofluorescence. (**e**) Quantitative statistical analyses of (**d**). Data are represented as means ± SEMs from triplicate experiments. If the same letter appears on the bars, the difference is not significant (*p* > 0.05). If there is no common letter among the bars, the difference is significant (*p* < 0.05). # *p* < 0.05, ## *p* < 0.01.

BoMac cells were pretreated with CPPs for 3 h and then exposed to BVDV for 48 h; the results showed notable differences in cell morphology and vitality. In the BVDV-infected group, cells appeared disrupted and largely detached, whereas cells pretreated with different doses of CPPs showed significantly alleviated damage (Figure 2a). The cell vitality assay indicated a significant reduction in cell vitality in the BVDV group (*p* < 0.05), but a notable improvement in cell vitality was observed following CPP pretreatment compared to the BVDV group (*p* < 0.05) (Figure 2b). Furthermore, the results revealed that the viral nucleic acid copy number and viral protein expression in the CPP-pretreated group were markedly reduced compared with the BVDV-infected group (*p* < 0.05) (Figure 2c–e). These findings indicated that CPPs effectively blocked BVDV adsorption to cells.

### 3.3. CPPs May Exert the Antiviral Effect by Directly Inactivating Virions

**Figure 3:**
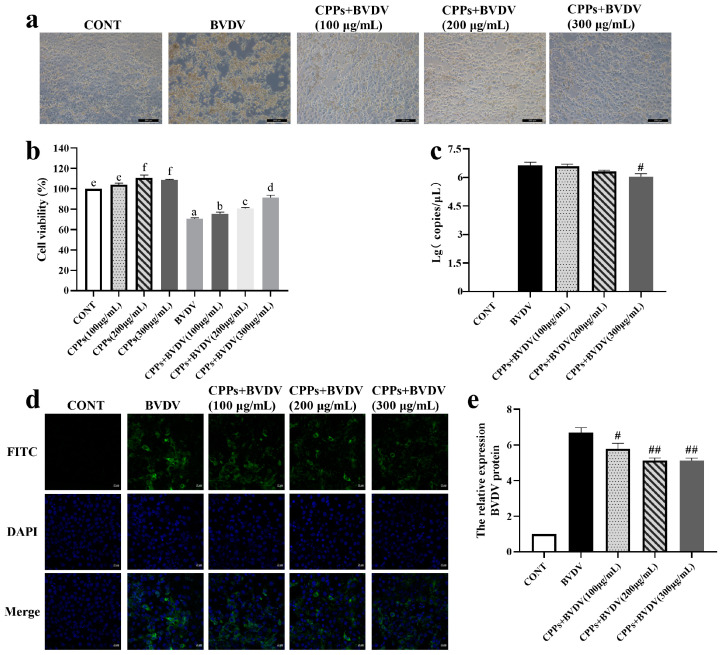
CPPs directly target virions. (**a**) Cell morphology changes were observed by inverted microscope. (**b**) Cell viability was determined by CCK-8 assay. (**c**) BVDV nucleic acid copy number was detected by RT-qPCR. (**d**) BVDV viral protein expression was detected by immunofluorescence. (**e**) Quantitative statistical analyses of (**d**). Data are represented as means ± SEMs from triplicate experiments. If the same letter appears on the bars, the difference is not significant (*p* > 0.05). If there is no common letter among the bars, the difference is significant (*p* < 0.05). # *p* < 0.05, ## *p* < 0.01.

To assess the direct killing effect of CPPs on BVDV particles, CPPs and BVDV were mixed thoroughly and incubated at 37 °C for 3 h before being added into cells for 48 h. Observations of cell morphology revealed that extensive cell disruption and detachment were exhibited in the BVDV group, whereas various doses of CPP treatment significantly alleviated cell damage, as shown in Figure 3a. The cell vitality assay results show a substantial vitality reduction in the BVDV group (*p* < 0.05), while the co-treatment group exhibited a marked improvement in cell vitality (*p* < 0.05) (Figure 3b). Analysis of the viral nucleic acid copy numbers indicates a significant reduction in only the high-dose-CPP group compared to the BVDV group (*p* < 0.05) (Figure 3c). Furthermore, viral protein was significantly reduced after co-treatment with CPPs and BVDV (*p* < 0.05) (Figure 3d,e). These findings suggest that CPPs exert a direct killing effect on BVDV, particularly effective at high CPP doses.

### 3.4. Blocking Effect of CPPs on BVDV Replication

**Figure 4:**
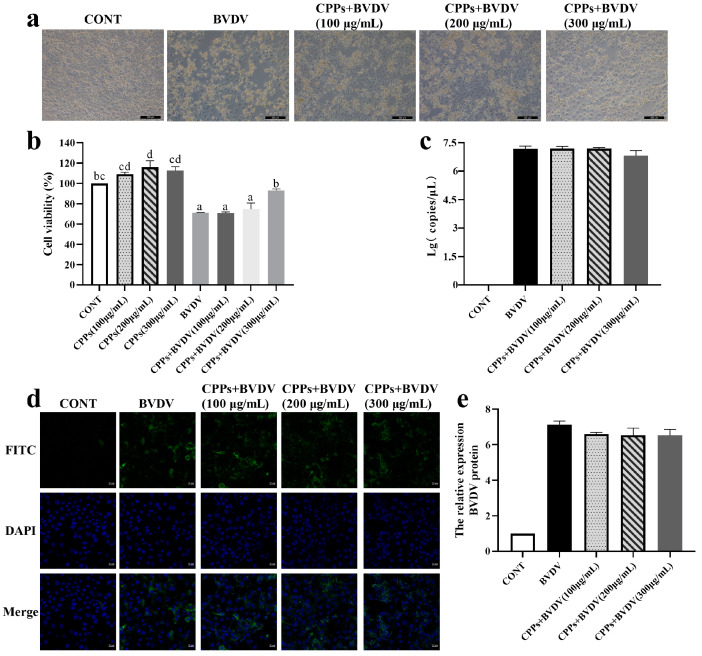
CPP inhibits the replication of BVDV in BoMac cells. (**a**) Cell morphology changes were observed by inverted microscope. (**b**) Cell viability was determined by CCK-8 assay. (**c**) BVDV nucleic acid copy number was detected by RT-qPCR. (**d**) BVDV viral protein expression was detected by immunofluorescence. (**e**) Quantitative statistical analyses of (**d**). Data are represented as means ± SEMs from triplicate experiments. If the same letter appears on the bars, the difference is not significant (*p* > 0.05). If there is no common letter among the bars, the difference is significant (*p* < 0.05).

BoMac cells were pretreated with BVDV for 3 h, followed by treatment with various doses of CPPs for 48 h. Observations of cell morphology indicate that extensive disruption and detachment were displayed in the BVDV group, whereas treatment with different CPP doses significantly mitigated cell damage (Figure 4a). The cell vitality assays demonstrated a marked reduction in vitality in the BVDV group (*p* < 0.05), while the CPP treatment resulted in a significant improvement in cell vitality compared to the BVDV group (*p* < 0.05) (Figure 4b). However, further examination of the viral nucleic acid copy number and viral protein expression revealed no significant changes in the CPP treatment group (*p* > 0.05), as shown in Figure 4c–e. This finding suggests that CPPs might not affect the replication of BoMac cells to BVDV.

### 3.5. Effects of CPPs on Cellular Phagocytosis Induced by BVDV in BoMac Cells

**Figure 5:**
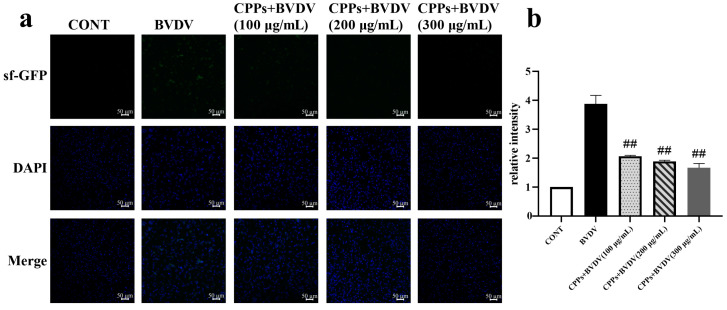
Effect of CPPs on phagocytic function in BVDV-infected BoMac cells. (**a**) Immunofluorescence assay of cell phagocytic function. (**b**) Quantitative statistical analyses of (**a**). Data are represented as means ± SEMs from triplicate experiments. ## *p* < 0.01.

Macrophages have a powerful phagocytic function, being able to engulf and digest viral particles, directly reducing the number of viruses. In this study, by using sf-GFP-labeled *E. coli* incubation, the effect of BVDV on BoMac cells’ phagocytic function and the regulatory role of CPPs were analyzed using laser confocal microscopy. The results show that positive fluorescence signals were observed inside the BoMac cells infected with sf-GFP-labeled *E. coli*, and the signals were higher in the BVDV group than in the control cells (*p* < 0.05); additionally, the signals were lower in the CPP-pretreated cells than in the BVDV group (*p* < 0.05) (Figure 5). This finding suggests that CPPs can enhance the phagocytic function of BVDV-infected BoMac cells.

### 3.6. Effects of CPPs on Antigen-Presentating Function Induced by BVDV in BoMac Cells

**Figure 6:**
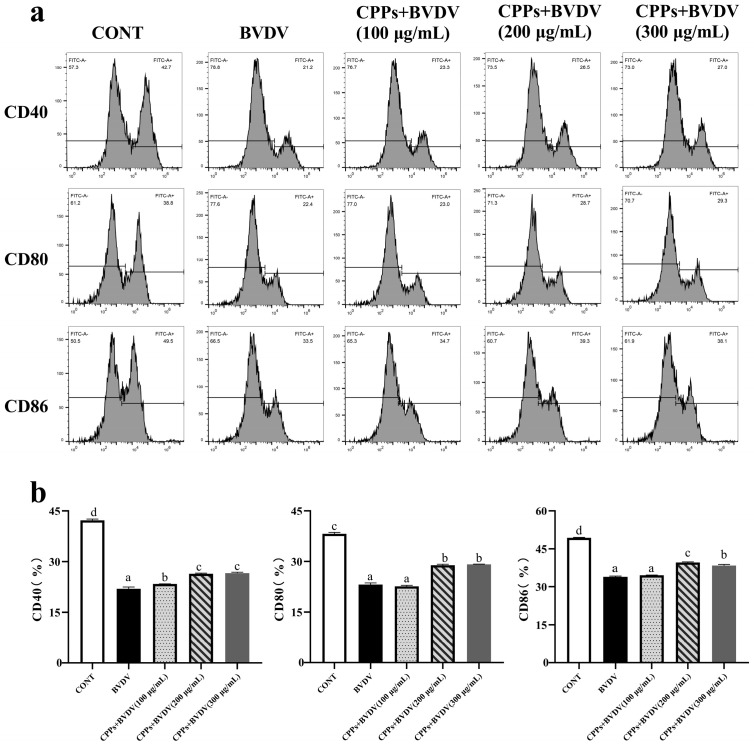
Effect of CPPs on the antigen-presenting function of BVDV-infected BoMac cells. (**a**) Flow cytometry analysis of the antigen-presenting function of cells. (**b**) Quantitative statistical analysis of (**a**). Data are represented as means ± SEMs from triplicate experiments. If the same letter appears on the bars, the difference is not significant (*p* > 0.05). If there is no common letter among the bars, the difference is significant (*p* < 0.05).

Macrophages can act as antigen-presenting cells, initiating specific immune responses to identify and eliminate virus-infected cells. Therefore, the antigen-presenting function was evaluated by detecting co-stimulatory molecules on the surface of BoMac cells through flow cytometry. The results reveal that the expressions of CD40, CD80, and CD86 on the cell surface were significantly reduced in the BVDV-infected group (*p* < 0.05). In contrast, the expressions of CD40, CD80, and CD86 were significantly increased upon pretreatment with 200 μg/mL and 300 μg/mL of CPPs, and only the expression of CD40 was significantly increased upon pretreatment with 100 μg/mL of CPPs (*p* < 0.05) (Figure 6). This finding suggests that CPPs can enhance the antigen-presenting function of BVDV-infected BoMac cells.

### 3.7. Effects of CPPs on the Expression of Inflammatory Factors Induced by BVDV in BoMac Cells

**Figure 7:**
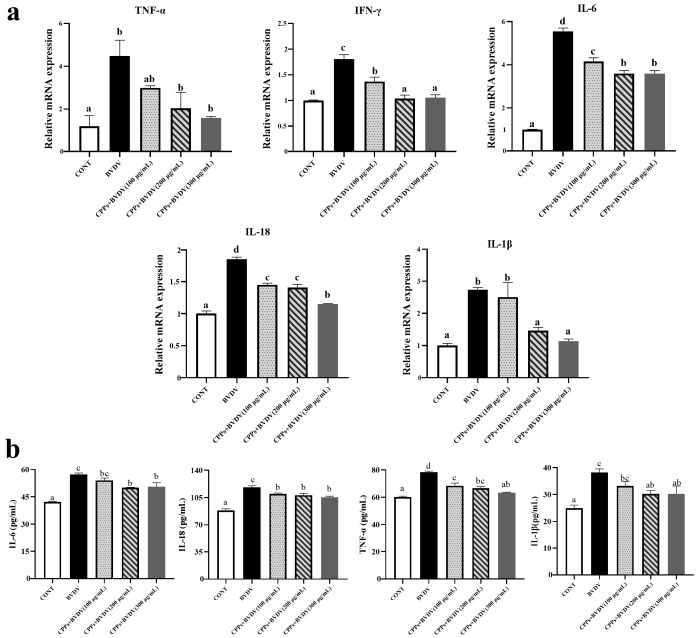
Effect of CPPs on the expression of inflammatory factors in BVDV-infected BoMac cells. TNF-α, IFN-γ, IL-6, IL-18, and IL-1β mRNA levels were quantified using RT-qPCR. IL-6, IL-18, TNF-α, and IL-1β protein expression levels were detected by ELISA. (**a**) Expression of TNF-α, IFN-γ, IL-6, IL-18, and IL-1β mRNA. (**b**) Protein expression of IL-6, IL-18, TNF-α, and IL-1β. Data are represented as means ± SEMs from triplicate experiments. If the same letter appears on the bars, the difference is not significant (*p* > 0.05). If there is no common letter among the bars, the difference is significant (*p* < 0.05).

Inflammatory-related cytokines play a key role in rapidly responding to pathogen infection and activating distinct immune cells. Therefore, ELISA and RT-qPCR methods were used to explore the consequences of CPPs on the BVDV-triggered expression of inflammatory cytokines. The results demonstrate that CPP administration significantly (*p* < 0.05) repressed the BVDV-induced genetic (TNF-α, IFN-γ, IL-6, IL-18, and IL-1β) (Figure 7a) and protein (IL-6, IL-18, TNF-α, and IL-1β) (Figure 7b) over-expression of inflammatory cytokines. These results demonstrate that CPPs inhibit the BVDV-induced inflammatory response.

### 3.8. Effects of CPPs on Cell Apoptosis Induced by BVDV in BoMac Cells

**Figure 8:**
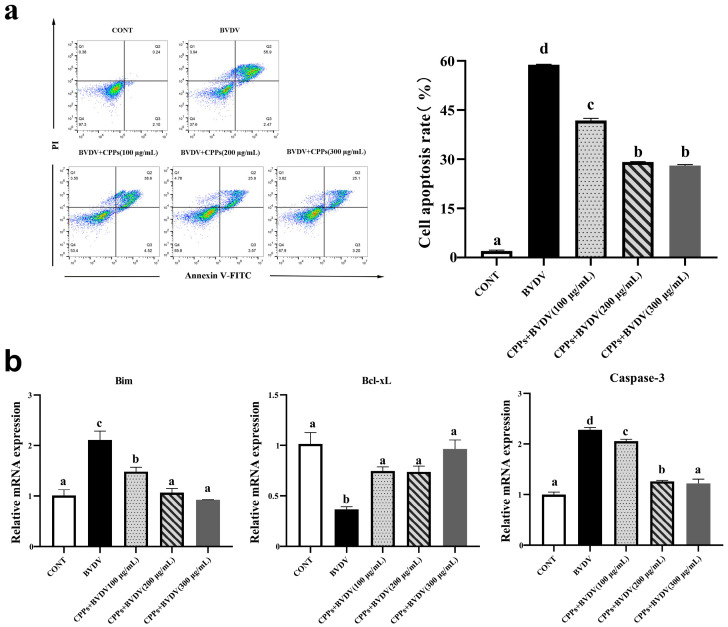
Effect of CPPs on apoptosis in BVDV-infected BoMac cells. Apoptosis rate was measured by flow cytometry, and Bim, Bcl-xL, and Caspase-3 mRNA levels were quantified using RT-qPCR. (**a**) Apoptosis rate. (**b**) mRNA expression of Bim, Bcl-xL, and Caspase-3. Data are represented as means ± SEMs from triplicate experiments. If the same letter appears on the bars, the difference is not significant (*p* > 0.05). If there is no common letter among the bars, the difference is significant (*p* < 0.05).

Viral infections can lead to apoptosis, thereby disrupting normal immune responses. To investigate the effects of CPPs on the apoptosis process triggered by BVDV infection in BoMac cells, flow cytometry was employed to analyze the cell apoptosis rate. The results show that additional CPPs pretreatment resulted in a lower cell apoptosis rate compared with the BVDV group (Figure 8a), and the mRNA expressions of the pro-apoptosis factors Bim and Caspase-3 were immensely (*p* < 0.05) increased in the BVDV group, and then decreased (*p* < 0.05) upon CPP pretreatment. The mRNA expression of the anti-apoptosis factor Bcl-xL was immensely (*p* < 0.05) decreased in the BVDV group, and then increased (*p* < 0.05) upon CPP pretreatment (Figure 8b). These results demonstrate that CPPs alleviate BVDV-induced apoptosis.

## 4. Discussion

BVDV can lead to immunosuppression and persistent infections, and current vaccines are ineffective against BVDV infection due to the antigenic variability of prevalent BVDV strains and subgenotypes [23]. Therefore, discovering and developing antiviral drugs is crucial for disease control except for the development of new BVDV vaccines. CP is a traditional Chinese medicine whose polysaccharide components have been shown to have antiviral and immunomodulatory properties [21,24]. However, there has not yet been any report on CPPs’ resistance to BVDV infection. Our study first confirmed that CPPs exerted antiviral effect against BVDV infection in BoMac cells, including adsorption blocking and direct inactivation. However, the direct inactivation of BVDV by CPPs was not as significant as the inhibition of BVDV adsorption by CPPs. Furthermore, we found that CPPs significantly enhanced cellular phagocytosis and antigen-presenting functions, as well as reducing the production of pro-inflammatory cytokines, apoptosis, and apoptosis-associated factors. These results confirm that CPPs may inhibit BVDV adsorption on BoMac cells by modulating immune response, thereby suppressing viral infection, which suggests that CPPs may play a potential role in BVDV defense and control.

The viral life cycle involves multiple complex steps, starting with the binding of viral proteins to cell surface receptors, followed by the fusion of the viral envelope with the cell membrane, and then replication in cells [25]. Antiviral drugs can intervene in various stages of this process to inhibit viral infection [26]. Previous studies have shown that *Rehmmannia glutinosa* polysaccharides typically inhibit viral infection by obstructing virus adsorption and exerting direct antiviral effects [27]. It was reported that plant and mushroom polysaccharides can exert antiviral effects by targeting the early stages of the viral life cycle, particularly attachment and entry [28]. In this study, CPPs were added at different stages to verify the effect of CPPs against BVDV infection. The results indicate that pretreatment with CPPs enhanced cell protection and prevented virus adsorption, and it is noteworthy that the high concentration of CPPs had a direct killing effect on BVDV, while a significant effect on viral replication blockade was not observed. Therefore, it can be concluded that the antiviral effects of CPPs on BVDV were mainly dominated by virus adsorption inhibition and direct antiviral effects, with virus adsorption blocking being the most obvious. Therefore, we used the mode of action of CPP pretreatment for subsequent experimental manipulations in this study.

BVDV infection can induce immunosuppression, which is associated with immune system evasion; therefore, viral infection activates macrophages to trigger a variety of immunomodulatory processes, including phagocytosis and antigen presentation [29,30]. Phagocytosis is a typical innate immune response in macrophages that contributes to the clearance of pathogens [31]; whilst acting as effective antigen-presenting cells, macrophages can present foreign antigens to T cells, accompanied by the expression of co-stimulatory molecules [32]. David et al. [33] found that influenza A virus can reduce macrophage phagocytosis. Luft et al. [34] also found that LCMV cl-13 can reduce macrophage surface CD80 and CD86 expressions. *Rubus chingii Hu* polysaccharides were confirmed to show activity that enhances macrophage phagocytosis as well as the expression of the surface co-stimulatory molecules CD40, CD80, and CD86 [35]. Consistent with the findings in our study evaluating the phagocytic and antigen-presenting functions of BoMac cells, BVDV infection significantly reduced phagocytosis as well as the expression of the co-stimulatory molecules CD40, CD80, and CD86 in BoMac cells. However, pretreatment with CPPs significantly improved phagocytosis and increased the expression of CD40, CD80, and CD86, suggesting that CPPs can stimulate the activation of BoMac cells, thereby attenuating BVDV-induced immune injury in BoMac cells.

The immune response communicates through cytokines to coordinate specific types of defense responses; therefore, cytokines play a crucial role in the immune response against pathogenic infections. In recent years, there has been increasing evidence that BVDV infection can induce an inflammatory response, which can lead to tissue and cellular damage in organisms [36]. Plant polysaccharides can regulate and balance excessive inflammation and related factors, and can reduce damage during viral infection [37,38,39]. Chen et al. [40] found that porcine circovirus type 2 viral infection induced an inflammatory response, and that *Sophora subprostrate* polysaccharides can play a preventive role in regulating the inflammatory response. In this study, we examined the expression of cytosolic inflammatory factors, and it was observed that BVDV infection significantly increased cellular inflammatory factors at the mRNA and protein levels. Pretreatment with CPPs significantly reduced the BVDV-induced mRNA and protein up-regulation. Our data further corroborate these studies, reinforcing the key role of plant polysaccharides in regulating inflammatory responses in the pathogenesis of BVDV.

Apoptosis is a highly regulated process that plays a crucial role in multicellular organisms by maintaining tissue homeostasis and eliminating damage or potentially harmful cells [41,42]. However, viruses can trigger excessive apoptosis in the late stages of replication to facilitate the release of progeny viral particles and accelerate the infection of neighboring cells [43], and these inflammatory factors can also directly or indirectly contribute to apoptosis [44]. Numerous studies have indicated that plant polysaccharides can down-regulate the expression of apoptosis markers such as Caspase-3, thereby inhibiting apoptosis [45,46]. In this study, we assessed the level of apoptosis, and the results show that BVDV infection significantly increased the apoptosis, with an elevation in pro-apoptotic factors’ mRNA expression and a decrease in anti-apoptotic factors’ mRNA expression. Pre-treatment with CPPs markedly reduced the apoptosis rate and regulated the expression of apoptosis-related factors. Our findings are consistent with these studies, demonstrating that CPPs can inhibit apoptosis, thereby suppressing viral release and mitigating cellular damage during viral infection. In conclusion, we found that CPPs may prevent BVDV infection of BoMac cells by modulating the immune response.

This study attempted to interpret the anti-BVDV effects of CPPs through immune regulation. The major limitation is that the immune regulatory mechanisms were not elucidated, and this study only used an in vitro model of BoMac cells. Therefore, we must highlight that this is a preliminary exploration of the antiviral effects of CPPs through immune regulation. Future research needs to establish in vivo infection models to further elucidate the protective effects of CPPs on animals.

## 5. Conclusions

In conclusion, we report for the first time the inhibitory effects of CPPs on BVDV infection in BoMac cells. CPPs have an inhibitory effect on the adsorption of viruses and a direct killing effect, especially during the BVDV adsorption stage. In addition, CPPs can reduce BVDV-induced inflammatory responses and apoptosis in BoMac cells through immunomodulation. These results suggest that in in vitro tests, CPPs inhibit BVDV infection of BoMac cells through immunomodulatory effects, and present CPPs as a potential treatment against BVDV infection. This study provides a theoretical basis for the in-depth development and utilization of *Codonopsis* resources and provides basic data on the development of anti-BVDV drugs. However, further exploration of the immune regulatory mechanisms of CPPs and in vitro tests are necessary.

## Figures and Tables

**Table 1:** Primers used for RT-qPCR.

Target Genes	Primer Sequence (5′-3′)	Product Length (bp)	Serial Number
TNF-α	F: AGGGGTCATGTGTGTGGAGAGC	99	NM_001166486.1
	R: GTGTGCAGGTGCCGGTTCAG		
IFN-γ	F: GAGCCTGTGAGCGTGCTTTT	163	NM_001077840.1
	R: TGGTGCTGAGGATGACATGG		
IL-6	F: GCCTTCACTCCATTCGCTGTC	109	NM_173923.2
	R: TGCCTGGGGTGGTGTCATT		
IL-18	F: TCATTAACCAGGGAAATCAACC	84	NM_174091.2
	R: TGATAAATATGGTCTGGGGTGC		
IL-1β	F: CCTCGGTTCCATGGGAGATG	119	NM_174093.1
	R: AGGCACTGTTCCTCAGCTTC		
Bim	F: AGCCCGGCACCCATGAGTTGT	215	XM_010809717.4
	R: GCCTGGTGACGCACGAAGACCCT		
Bcl-xL	F: CGACGGGCATTCAGCGACCT	122	XM_005214498.5
	R: GCCACAATGCGACCCCAGTTCACC		
Caspase-3	F: CTGGACCCTCATCCATCCTT	168	NM_001077840.1
	R: GGCACCCTGGTTCTTTCATTT		
β-actin	F: GCGGCATTCACGAAACTACCTT	268	NM_173979.3
	R: TCCTGCTTGCTGATCCACATCT		

## Data Availability

The original contributions presented in the study are included in the article; further inquiries can be directed to the corresponding authors.

## Abbreviations

The following abbreviations are used in this manuscript:

BVD	Bovine viral diarrhea
BVDV	Bovine viral diarrhea virus
CPPs	*Codonopsis pilosula* polysaccharides
BoMac	Bovine macrophage
CP	*Codonopsis pilosula*
MDBK	Madin-Darby bovine kidney
FITC	Fluorescein isothiocyanate
MOI	Multiplicity of infection
OD	Optical density
RT-qPCR	Real-time quantitative PCR
ELISA	Enzyme-linked immunosorbent assay
*E. coli*	*Escherichia coli*
SEMs	Standard errors of means
